# Exploring opportunities for drug repurposing and precision medicine in cannabis use disorder using genetics

**DOI:** 10.1111/adb.13313

**Published:** 2023-07-14

**Authors:** Laura A. Greco, William R. Reay, Christopher V. Dayas, Murray J. Cairns

**Affiliations:** ^1^ School of Biomedical Sciences and Pharmacy The University of Newcastle Callaghan New South Wales Australia; ^2^ Precision Medicine Research Program Hunter Medical Research Institute New Lambton New South Wales Australia

**Keywords:** Cannabis use disorder, Genetics, Pharmacotherapy

## Abstract

Cannabis use disorder (CUD) remains a significant public health issue globally, affecting up to one in five adults who use cannabis. Despite extensive research into the molecular underpinnings of the condition, there are no effective pharmacological treatment options available. Therefore, we sought to further explore genetic analyses to prioritise opportunities to repurpose existing drugs for CUD. Specifically, we aimed to identify druggable genes associated with the disorder, integrate transcriptomic/proteomic data and estimate genetic relationships with clinically actionable biochemical traits. Aggregating variants to genes based on genomic position, prioritised the phosphodiesterase gene *PDE4B* as an interesting target for drug repurposing in CUD. Credible causal *PDE4B* variants revealed by probabilistic finemapping in and around this locus demonstrated an association with inflammatory and other substance use phenotypes. Gene and protein expression data integrated with the GWAS data revealed a novel CUD associated gene, *NPTX1*, in whole blood and supported a role for hyaluronidase, a key enzyme in the extracellular matrix in the brain and other tissues. Finally, genetic correlation with biochemical traits revealed a genetic overlap between CUD and immune‐related markers such as lymphocyte count, as well as serum triglycerides.

## Graphical abstract

The prevalence of cannabis use disorder is increasing; however, there remain no approved medications for the treatment of this condition. We leveraged data from the largest genetic study of cannabis use disorder to date to prioritise treatment targets for the disorder.

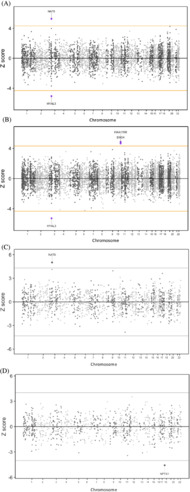

## 1 INTRODUCTION

Cannabis is currently one of the most widely used psychoactive substances worldwide, with an estimated 200 million users in 2018 (UNODC World Drug Report 2020)^1^. However, one in five users meet diagnostic criteria for CUD,^2^ which is characterised by increased compulsivity, physical dependence and difficulty achieving abstinence.^3^ Despite these issues, the validity of cannabis dependence and withdrawal was previously not well recognised. This can be seen through the Global Burden of Disease (GBD) excluding cannabis dependence in 1990^4^ and cannabis withdrawal not being included in the DSM until the Fifth Edition in 2013 and International Classification of Diseases until the Tenth Revision (ICD‐10) in 2015.^5^

The largest published CUD genome‐wide association study (GWAS) to date was performed by Johnson et al in 2020.^6^ This study identified two genome‐wide significant loci on chromosome 7 (*FOXP2*) and chromosome 8 (near *CHRNA2* and *EPHX2*), with an estimated liability scale SNP‐heritability (h^2^_SNP_) for CUD, depending on the estimated population prevalence, as 6.7%–12.1%.^6^ Twin studies have provided insight into the total heritability (h^2^) of cannabis use and dependence, with the h^2^ for lifetime cannabis use (ever vs. never) shown to be around 45% and cannabis dependence as high as 78%.^7^, ^8^ The high heritability of CUD suggests a strong genetic component, which could be leveraged to inform new treatment options. Drug repurposing, where existing compounds are used in a new indication, is an attractive option to expediate changes in CUD clinical practice, as investment and success in de novo drug development for psychiatry remains comparatively limited.

Despite the potential of genetics, the tremendous heterogeneity among individuals with CUD makes current drug development challenging. To date, no pharmacotherapy has been clearly effective, and there are no approved medications for CUD treatment.^5^, ^9^ Various pharmacological approaches have been tested to assist people with CUD in reducing their cannabis use by addressing withdrawal symptoms, craving and other cognitive factors. However, most of these interventions have not progressed beyond small pilot trials.^10^, ^11^

Brezing and Levin's report highlights the need to consider individual patient characteristics for the treatment of CUD. Complex, polygenic disorders such as CUD do not have a one‐size‐fits‐all approach,^5^ and research over the past 20 years has shown that not everyone who uses cannabis is affected adversely in the same way. Emerging vulnerability factors, including certain genes and personality characteristics, are being identified, although the mechanisms underlying the negative effects of cannabis use are not fully understood.^12^ Given the heritability of CUD, genetics may provide a means to identify and prioritise novel treatment opportunities with greater specificity. Gene‐based enrichment approaches, functional genomics and genetic correlation and causality analyses could be used to identify and refine opportunities for pharmacological interventions. Furthermore, genetic analyses of therapeutically actionable traits such as serum blood biomarkers could also inform drug repurposing opportunities.^13^ In the present study, we explore these approaches to inform drug repurposing opportunities, expand on current literature and identify new therapeutic targets for those with CUD.

## 2 MATERIALS AND METHODS

### 2.1 GWAS

GWAS of cannabis use disorder (CUD) summary statistics on unrelated genotyped individuals of European ancestry (*N*_*cases*_ *= 14 080*, *N*_*controls*_ *=* 343 726) were obtained from the Psychiatric Genomics Consortium (PGC; https://pgc.unc.edu). These summary statistics are the combination of samples from the PCG Substance Use Disorders working group, iPSYCH and deCODE. The iPSYCH cohort consists of individuals born in Denmark between 1981 and 2005. CUD cases were defined using ICD10 codes (F12.1‐12.2).^14^ Controls were individuals who did not have ICD10 codes related to CUD. DeCODE cases were drawn from the largest addiction treatment centre in Iceland, the SAA‐National Centre of Addiction Medicine. CUD diagnoses in this treatment cohort were made by clinicians using the Diagnostic and Statistical Manual of Mental Disorders (DSM) system (DSM‐IIIR, DSM‐IV and DSM‐5 criteria).^15^ The cases in the PGC Substance Use Disorders working groups all met DSM‐IV diagnostic criteria.

### 2.2 Gene‐based analyses to identify druggable targets associated with CUD

Gene‐based association analysis for CUD GWAS summary statistics^6^ was performed using MAGMA version 1.09 (https://ctg.cncr.nl/software/magma). MAGMA maps SNPs to genes by aggregating common variants (MAF > 0.01) at the gene level, which increases discovery power by using the linear combination of SNP‐wise *P*‐values as test statistic, reducing the burden of multiple testing correction seen in univariate GWAS. We used the 1000 genomes phase 3 European reference panel population for LD estimation and mapped variants to the NCBI hg19 genome assembly, which contained 18 297 autosomal protein‐coding genes with SNPs mapped within the defined coordinates of 5 kb upstream and 1.5 kb downstream. Any genes from the major histocompatibility complex (MHC, chr6:28477797–33448354) on chromosome 6 were removed. Multiple testing correction was done using the Bonferroni method, where we divided the alpha level by the number of comparisons and set *P* < 2.7 × 10^−6^ as the *P*‐value required for significance.

### 2.3 Probabilistic finemapping

Given the association signal may be therapeutically actionable and to further support the MAGMA findings that *PDE4B* as the most significant gene in this locus, we subjected the *PDE4B* region to further analysis to evaluate whether it is a causal association. Firstly, we leveraged probabilistic finemapping to prioritise putatively causal genetic variation in this region. Specifically, we used a conventional Bayesian approach that was applied to all variants in the CUD GWAS within 3 MB of the defined *PDE4B* genic boundaries. Asymptotic Bayes' factors (ABF) for each SNP were approximated using Wakefield's method, assuming a prior variance of 0.2,^2^ as outlined extensively elsewhere.^16^ ABF were summed to define credible sets given that Bayes' factors are proportional to the posterior probability (*PP*) for causality for each variant. In other words, to define a 95% credible set of variants in this region, which contains a causal variant with 95% probability, variant‐wise *PP* were summed in descending order until 0.95 is exceeded. This method assumes a single causal variant so that we did not have to account for LD between variants, which has been demonstrated to be susceptible to false positives in finemapping studies with a prior of multiple causal variants that use references external to the GWAS or not otherwise very well matched at a population level.^17^

### 2.4 *PDE4B* phenome‐wide association study

We then wished to further investigate the phenotypic relevance for the finemapped CUD association signal in *PDE4B*. For the variant with the highest *PP*, we performed a phenome‐wide association study (pheWAS) using IEU open GWAS project database (https://gwas.mrcieu.ac.uk/phewas/) and the FinnGen release 7 resource (https://r7.finngen.fi/about). The concept underlying this is to assess other traits to which this variant is linked.

### 2.5 Colocalisation of the *PDE4B* association signals with expression quantitative trait loci

We then attempted to infer using expression quantitative trait loci (eQTL) data whether upregulation or down‐regulation of *PDE4B* was associated with liability to CUD and whether CUD and *PDE4B* expression displayed statistical colocalisation. *PDE4B* eQTLs were sourced from the multi‐tissue eQTL catalogue resource (https://fivex.sph.umich.edu/).^18^ We used cortical eQTL data assembled by the MetaBrain consortium (*N* = 2970) for colocalisation analyses via the *coloc* method.^19^ The *coloc* approach infers the *PP* of five competing hypotheses (*H*) for a given region: *H*_0_ = the region is associated with neither trait, *H*_1_ = the region is associated with trait one, *H*_2_ = the region is associated with trait two, *H*_3_ = the region is associated with both traits but with a different underlying causal variant, and *H*_4_ = both traits share a causal variant. This was implemented using default priors via version 4 of the coloc R package. We also performed a sensitivity analysis where we varied the SNP priors between (1 × 10^−8^ and 1 × 10^−4^), as implemented by the *sensitivity* function in the coloc package.

### 2.6 Functional genomics analyses of CUD using transcriptome‐ and proteome‐wide association studies

To further refine our understanding of CUD associated genes that may present opportunities for repurposing, we performed a transcriptome‐wide association study (TWAS) and a proteome‐wide association study (PWAS) via the FUSION framework.^20^ We achieved this through leveraging genetically imputed models of mRNA and protein expression. For TWAS, we used tissue samples of whole blood (GTEx v7) and post‐mortem brain (GTEx v7, PsychENCODE),^20^, ^21^ whereas protein expression weights of post‐mortem brain and whole blood were yielded from Religious Orders Study and Memory and Aging Project (ROS/MAP)^22^ and NHLBI's Atherosclerosis Risk in Communities (ARIC),^23^ respectively. As this method integrates SNP effects from the model of genetically predicted expression with the effects of the same SNPs on CUD, TWAS/PWAS Z‐scores, after accounting for linkage disequilibrium (LD), can be a conceptualised measure of genetic covariance between mRNA or protein expression of the gene and the GWAS trait of interest. As a result, the sign of the TWAS/PWAS *Z* score is informative as to which direction of genetically predicted mRNA or protein expression is associated with increased odds of CUD. To ensure we captured only the most confidently associated genes that could constitute drug repurposing candidates, we utilised a conservative method for multiple‐testing correction whereby the Bonferroni methodology was implemented to divide the alpha level (0.05) by the total number of significantly cis‐heritable models of genetically regulated expression (GReX) tested from any brain tissue considered or whole blood. Finally, we implemented probabilistic finemapping of TWAS Z scores using FOCUS v0.6.10 to refine potential causal genes for which predicted expression is associated with CUD, as outlined extensively elsewhere.^24^, ^25^ Using the default prior and prior variance, we estimated marginal posterior inclusion probabilities (*PIP*) for membership of 90% credible set for each gene in the region in and around the implicated hyaluronidase gene cluster given its potential therapeutic relevance.

### 2.7 Genetical correlation between CUD and biochemical traits

LD‐score regression analysis (LDSR; v1.0.1) (https://github.com/bulik/ldsc)^26^ was used to estimate genetic correlation between CUD and GWAS on 50 biochemical traits from the UK Biobank (UKBB) as outlined previously by our group for other psychiatric disorders.^13^ As the mode of action of many existing drugs involves modulating biochemical traits, for example, lipids and blood glucose, shared biology that may be indexed by genetic correlation could be informative as drug repurposing opportunities. In LDSR, the genetic covariance is estimated by regressing SNP‐wise χ^2^, the product of the marginal SNP effects from both traits, on its LD score. The SNP heritability estimates for both traits are used to normalise the genetic covariance to obtain genetic correlation (*r*_g_), which can be accurately estimated in the presence of any sample overlap only affects the LDSR intercept and not the slope. Bonferroni multiple testing correction was used for the 50 biochemical traits tested to define a significant r_g_. The CUD and biochemical trait GWAS summary statistics were cleaned to ensure they contain HapMap3 SNPs outside the MHC with minor allele frequency >0.05 for consistency. To evaluate evidence of a causal relationship, we used the latent causal variable (LCV) on all genetically correlated CUD and biochemical trait pairs as demonstrated elsewhere and in our previous work.^13^, ^27^ To estimate partial genetic causality, the LCV framework leverages the bivariate genome‐wide distribution of marginal SNP effects on both CUD and each of the biochemical traits and outputs the posterior mean genetic causality proportion metric (*GCP*), with *GCP* > 0 implying partial genetic causality of trait one on trait two, and vice versa. To calculate the *GCP* metric, the LCV model utilises the genome‐wide SNP–trait association Z scores for two traits and the mixed fourth moments (cokurtosis) of the respective distributions to assess whether there is evidence for a causal effect of one trait on the other. To guard against false positives, partial genetic causality was defined using the recommended threshold of a significantly non‐zero|*GCP*| > 0.6.^27^

## 3 RESULTS

### 3.1 Exploring drug interactions of CUD‐associated genes

Using aggregated gene‐level association (MAGMA), we observed two genes that were associated with CUD—*PDE4B* (*P =* 2.09 × 10^−6^) and *FOXP2* (*P =* 9.30 × 10^−7^) after Bonferroni correction (*P* < 2.7 × 10^−6^; Table S1). These genes have been previously reported in Johnson et al.^6^ Further analysis using the Drug Gene Interaction Database (DGIdb) on the MAGMA significant genes for CUD revealed the highest interaction score for *PDE4B* was with dyphylline, a vasodilator and bronchodilator agent. Dyphylline is a phosphodiesterase 4 inhibitor clinically used for the prevention of bronchial asthma or other respiratory diseases.^28^ It has similar pharmacological actions and safety profiles as other xanthine derivatives, such as caffeine and theobromine.^29^ According to DrugBank, the mechanism of action of dyphylline in humans is a cAMP‐specific 3′,5′‐cyclic phosphodiesterase 4A, 4B, 4C and 4D inhibitor, and an adenosine receptor A1 and A2a antagonist.^30^, ^31^, ^32^, ^33^ No drug interactions for *FOXP2* were observed in DrugBank or in DGIdb. Interestingly, the *PDE3* inhibitor, dipyridamole, also targets *PDE4B* and has been investigated therapeutically for bipolar disorders.^34^

Using probabilistic finemapping, we prioritised one variant in the 95% credible set with a posterior probability greater than 40% (rs1392816, *PP* = 0.534), out of a total of 34 variants within the credible set. This variant was an intronic variant in the *PDE4B* gene but was not associated with *PDE4B* mRNA expression in any of the eQTL catalogue studies or in the MetaBrain cortical eQTL dataset. A pheWAS in the IEU GWAS database of this variant revealed that it was associated with mostly inflammatory [e.g. leukocyte counts, C‐reactive protein (CRP)] and anthropometric (e.g. fat mass, waist‐to‐hip ratio) measures using a conventional phenome‐wide significance threshold (*P* < 1 × 10^−5^) (Tables S2 and S3), However, it also demonstrated an association with smoking behaviours. In the FinnGen GWAS database, which only considers binary phenotypes collected from linked hospital inpatient records, it was associated with alcohol use disorder and other related substance abuse phenotypes, supporting its phenotypic relevance for CUD. Statistical colocalisation between *PDE4B* expression in the cortex and CUD supported the third colocalisation hypothesis that this region is associated with both *PDE4B* expression and CUD, but both traits are influenced by a different causal variant (*PP*_*H3*_ = 0.93). This conclusion remained consistent with different priors. Indeed, there was no strong evidence to support the CUD‐associated variants mapped to *PDE4B* as acting as eQTLs, suggesting that the effect of genetic variation in this region is mediated through another modality or it influences expression in a tissue or cell type not considered.

### 3.2 Dysregulated hyaluronidase enzyme expression associated with liability to CUD through integration of transcriptomic and proteomic data

**FIGURE 1:**
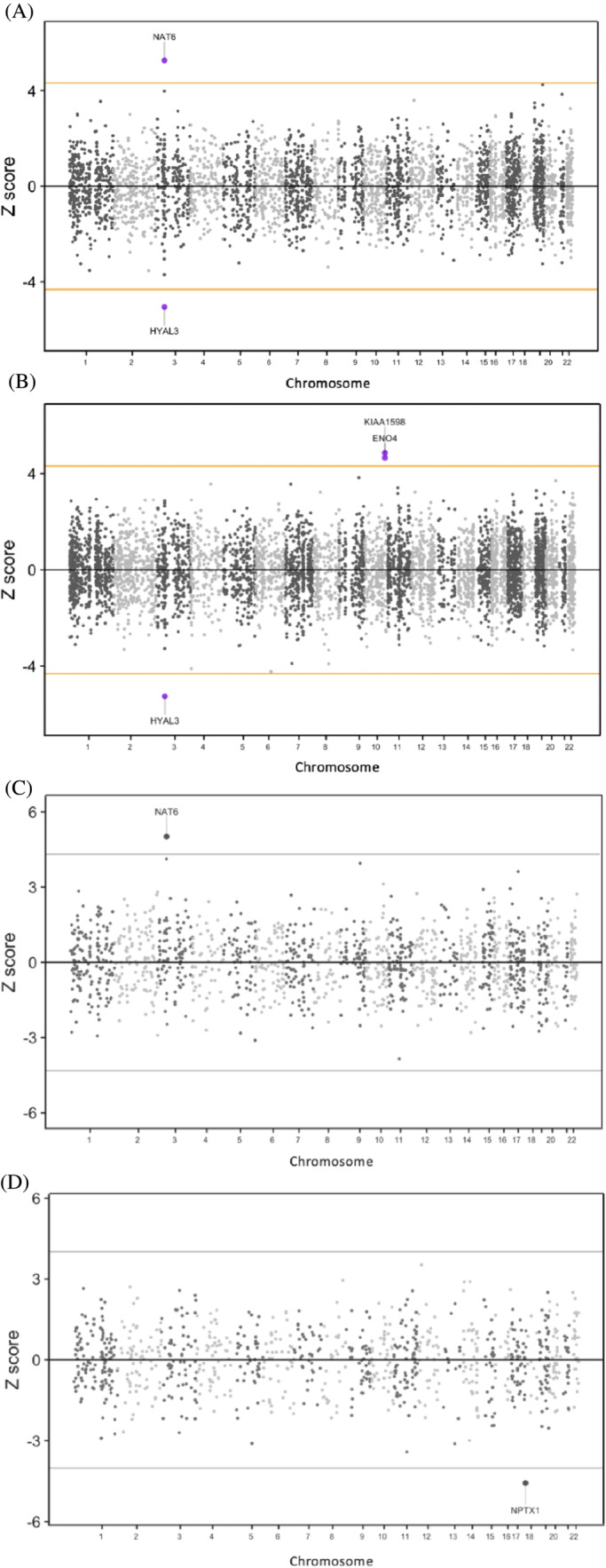
Miami plot of cannabis use disorder (CUD) transcriptome‐wide association (TWA) analysis of brain cerebral cortex (A) and whole blood (B) and proteome‐wide association (PWA) analysis of brain (C) and whole blood (D). The orange horizontal lines are the significance threshold after Bonferroni correction for the total number of imputated models of expression (alpha/n).

We utilised TWAS and PWAS methods to integrate genomic information into functionally relevant units that map to genes or proteins and their expression. Next, we assessed the statistical associations between CUD and predicted gene expression. Although TWAS/PWAS associations do not necessarily imply a causal relationship, this approach can be implemented to identify candidate genes located at loci with an inferred mechanistic underpinning through expression.^35^ In multi‐brain region and blood TWAS of CUD, significantly down‐regulated genetically proxied expression of the hyaluronidase gene *HYAL3* was associated with the disorder in the whole blood, as well as several brain regions including the amygdala (*Z =* −4.294, *P =* 1.75 × 10^−5^), anterior cingulate cortex (*Z =* −5.103, *P =* 3.34 × 10^−7^), hippocampus (*Z =* −5.103, *P =* 3.34 × 10^−7^), hypothalamus (*Z =* −5.185, *P =* 1.05 × 10^−7^), nucleus accumbens (*Z =* −5.552, *P =* 2.82 × 10^−8^) and putamen (*Z =* −5.433, *P =* 5.51 × 10^−8^). The TWAS also revealed significantly up‐regulated expression of *NAT6*, also known as *HYAL1*, in the cortex (Figure 1A).

*NAT6* is a hyaluronidase that is proximally located on chromosome 3,^36^ similar to *HYAL3*. However, *HYAL1* is considered to be a more canonically active hyaluronidase compared to *HYAL3*.^37^, ^38^ These two transcripts were previously reported in the Johnson et al CUD GWAS using a different statistical method for TWAS (S‐PrediXcan). Additionally, a third gene *TTC3* was identified using expression weights from the 3p21.3 putamen region (*Z =* 4.31, *P =* 1.63 × 10^−5^). Overexpression of *TTC3* has been associated with negative effects on cognitive function and neuronal health.^39^, ^40^ As a result, this 3p21.3 region contains several plausible CUD risk genes, and future in silico and experimental follow‐up is warranted to disentangle true effects. In whole blood, *ENO4* (*Z =* 4.8618*, P =* 3.26 × 10^−6^) and *KIAA1598* (*Z =* 4.8618, *P =* 1.16 × 10^−7^), genes previously reported from European gene‐wise association analysis, were shown to be up‐regulated (Figure 1B). *KIAA1598* is also known as *SHTN1,* with Shootin‐1 protein isoform switch from long isoform (Shtn1L) to short isoform (Shtn1S) known to play an important role in axonogenesis^41^, ^42^ (Table S4).

We expanded our analysis beyond mRNA expression by conducting a PWAS. In the brain, we again found that *NAT6/HYAL1* was significantly up‐regulated in terms of protein abundance (*Z =* 5.019*, P =* 5.19 × 10^−7^) (Figure 1C). In the whole blood, we observed a novel significant association between predicted protein expression of *NPTX1* and CUD (*Z =* −4.57, *P =* 4.88 × 10^−6^), which is critical for early human development^43^ (Figure 1D). Although *NPTX1* down‐regulation was also detected in the TWAS, it did not reach statistical significance after correction for mRNA expression (Table S5).

### 3.3 Exploring the therapeutic potential of hyaluronidase‐1 enzyme inhibition

The TWAS/PWAS analyses revealed *HYAL1* as the most promising target for drug repurposing, as it can be modified by some existing nutraceuticals and investigational compounds (Table 1). However, the other TWAS/PWAS associations did not have current approved therapies that target them, although *PDE4B* (from the MAGMA analyses) is an existing drug target. If hyaluronidase dysregulation is indeed associated with CUD, as suggested by the TWAS/PWAS, it may represent a promising opportunity for therapeutic investigation. Hyaluronic acid, also known as hyaluronan, is a crucial component of the extracellular matrix and is known to play a role in the regulation of inflammatory processes and embryonic development.^44^ In the central nervous system (CNS), hyaluronic acid is a major component of perineuronal nets. These are mesh‐like structures that form around specific neuronal cell bodies and proximal dendrites and have a crucial role in synaptic stabilisation and plasticity.^45^ An increase in the expression of *NAT6/HYAL1*, which is associated with CUD, suggests that there may be abnormal breakdown of hyaluronic acid in individuals with this disorder, as hyaluronidases catalyse the degradation of hyaluronic acid. This abnormal breakdown could potentially increase the risk of developing CUD if the *NAT6/HYAL1* gene has a direct causal effect. However, this interpretation is conflicted by the association we found between down‐regulation of another proximal hyaluronidase, *HYAL3*, and CUD. This requires further investigation, although there is evidence *HYAL3* does not directly contribute to the metabolism of hyaluronic acid,^37^ but rather it is believed to do so by augmenting the activity of *HYAL1*.^38^ If hyaluronic acid catabolism were dysregulated in CUD, this finding may suggest the potential use of oral hyaluronic acid supplementation, which has been previously trialled for conditions like osteoarthritis. Additionally, the use of ascorbyl palmitate (L‐ascorbic acid 6‐hexadecanoate), an antioxidant that also inhibits hyaluronidase activity, may have therapeutic benefits as a neuroprotective agent. However, it is important to note that ascorbyl palmitate is not currently registered with the Food and Drug Administration (FDA) for therapeutic use (Table 1).^46^, ^47^, ^48^

**TABLE 1:** Brief overview of compounds under investigation for inhibition of hyaluronidase‐1.

Name	IC_50_	
Ascorbyl palmitate	4.2 ± 0.13 (SagHL)^46^	Potent hyaluronidase inhibitor in vitro. Esterified form of vitamin C.^46^ Globally approved antioxidant food additive (E304)
Chebulanin	132 μM (EcH1)^49^	Anti‐inflammatory and anti‐arthritic agent that inhibits NF‐κB and MAPK signalling pathway activation^50^, ^51^
Chicoric acid	171 μM (EcH1)^52^	Dicaffeoyl ester with properties that include anti‐inflammatory and anti‐aging properties and glucose and lipid metabolism regulation^53^
Glycyrrhizic acid	177 μM (EcH1)^54^	Known anti‐allergic, anti‐viral and anti‐inflammatory, anti‐lipidaemic and anti‐hyperglycaemic properties.^55^ FDA‐approved food additive
Testosterone propionate	124 ± 1.1 μM (EcH1)^52^	Slow‐release anabolic steroid. Shown to be neuroprotective in animal models of Parkinson's disease^56^

Abbreviations: EcH1, *Escherichia coli* F470 cells expressing Hyal1; SagHL, *Streptococcus agalactiae* hyaluronate lyase.

To attempt to resolve the role of hyaluronic acid catabolism in CUD via effects at this locus, we applied probabilistic finemapping of TWAS test statistics on the region located at 3p21.3 that harbours both *HYAL1* and *HYAL3*. Using a combined database of SNP weights from all GTEx tissues, as well some brain and blood datasets, *HYAL1* was prioritised as the most likely causal gene for CUD, with a *PIP* of being in the 90% credible set of 89.3%, compared to 1.51% for *HYAL3*. However, probabilistic finemapping applied to the PsychENCODE SNP weight set, wherein *HYAL1* did not have a model of genetically predicted expression available, prioritised *HYAL3* instead (*PIP* = 78.3%). Given this disparity, further work is still required to reconcile how hyaluronidase activity may be involved in the aetiology of CUD.

### 3.4 CUD genetically correlated with clinically significant metabolites and immune markers

We tested the genetic correlation between CUD and a panel of blood‐based biomarkers from the UKBB to gain further insight into drug repurposing opportunities by exploring the interplay between circulating biochemical factors and the pathophysiology of CUD. After Bonferroni correction, we found that CUD was genetically correlated with 12 of the 50 blood‐based biomarkers we tested. This included alanine aminotransferase (*r*_*g*_ *=* 0.185, *SE =* 0.034, *P =* 7.25 × 10^−7^), CRP (*r*_*g*_ *=* 0.206, *SE =* 0.55, *P =* 2.0 × 10^−4^), eosinophil count (*r*_*g*_ *=* 0.122, *SE = 0.032*, *P =* 9.75 × 10^−5^), gamma glutamyltransferase (*r*_*g*_ *=* 0.193, *SE = 0.043*, *P =* 7.94 × 10^−6^), lymphocyte count (*r*_*g*_ *=* 0.178, *SE =* 0.033, *P =* 6.04 × 10^−8^), triglycerides (*r*_*g*_ *=* 0.146, *SE =* 0.039, *P =* 2.0 × 10^−4^) and white blood cell (WBC) count (*r*_*g*_ *=* 0.155, *SE = 0.032*, *P =* 1.89 × 10^−6^). It is worth nothing that inflammation of the CNS has long been linked to psychiatric disorders, including schizophrenia.^57^ Although we did not find direct evidence of a causal effect, the shared biology between CUD and the biomarkers we tested using LDSC is still informative for future treatment opportunities (Tables S6 and S7).

## 4 DISCUSSION

CUD has become the primary use disorder for which individuals seek treatment for globally, surpassing all other substances (UNODC, 2017). In this study, we used genetic approaches to explore drug repurposing opportunities for CUD, which currently has no approved treatments. Using gene‐level association, we observed that SNPs localising at *PDE4B* had the greatest association with CUD. The causal variant in *PDE4B*, which was found using probabilistic finemapping and is associated with inflammation and substance use in a phenome‐wide analysis, is also believed to have a role in modulating the immune response of monocytes and neutrophils, given that *PDE4B* is the predominant isoform of the PDE4 protein family.^58^, ^59^ A role for inflammation in CUD is further supported by its positive genetic correlation with inflammatory markers like CPR and leukocyte count, although a causal relationship could not be confirmed explicitly in LCV models. This suggests that a more complex relationship may exist, perhaps through specific cytokine repertoires that were not able to be considered in this study. While these genetic correlations with CUD are not necessarily causal, they provide insight into potential shared biological mechanisms between biochemical traits that are targeted by drugs that warrant further investigation.

It is also plausible that *PDE4B* influences CUD more specifically in the brain given its high expression in the tissue. Inhibitors of type 4 phosphodiesterase (*PDE*) are known to have anti‐inflammatory effects in various cells, including glia, by increasing cAMP and reducing inflammatory signalling^60^. One such PDE inhibitor, ibudilast, can cross the blood–brain barrier and suppresses TNF‐alpha production and astrocyte and microglial activation, making it a potential treatment option for CUD.^61^, ^62^, ^63^

We also conducted PWAS analyses that suggested that increasing genetically predicted neuronal pentraxin 1 (*NPTX1*) protein expression was protective for CUD. As a member of the pentraxin superfamily, NPTX1 shares structural homology with CRP.^64^ *NPTX1* is predominantly expressed in the brain and plays an important role in the regulation of synaptic strength and plasticity, as well as neurodegeneration.^65^, ^66^ Abnormal expression of neuronal pentraxins has been linked to various psychiatric disorders, including schizophrenia and bipolar disorder.^67^, ^68^ Interestingly, *NPTX1* is a negative regulator of excitatory synapses, and its knock‐down has been shown to increase the number of excitatory synapses, while elevated levels of *NPTX1* in the plasma are linked to mild cognitive impairment.^69^, ^70^ If increased *NPTX1* expression is protective for CUD, this could reflect a compensatory mechanism in response to the increased number of excitatory synapses in the brain, as previous research has shown that administration of addictive drugs enhances excitatory synaptic strength.^64^ Further studies are needed to determine the causal relationship between *NPTX1* expression and CUD, as well as to investigate the potential therapeutic implications of targeting *NPTX1* in CUD treatment.

In this study, we extended the previously implicated role of *HYAL1* and *HYAL3* in CUD^6^ by demonstrating that an increased genetically proxied protein expression of *HYAL1* plausibly increases the risk for CUD beyond a mere association with mRNA expression. Our finemapping analyses provided some support that *HYAL1*, one of the two proximally located hyaluronidase genes, is more likely a candidate causal gene, although this was not definitive. The role of *HYAL1* is particularly interesting because it down‐regulates the expression of hyaluronic acid. A lack of hyaluronic acid in the brain has been shown to cause a reduction in extracellular space (ECS) volume and induce an epileptic phenotype in mice.^71^, ^72^ This effect could be because *HYAL1* digests high molecular weight hyaluronic acid into low molecular weight fragments intracellularly, and these small fragments are important for activating pathways involved in endothelial cell proliferation, adhesion and migration.^73^

In addition to the repurposing opportunities provided by oral hyaluronic acid itself or the hyaluronidase inhibitor ascorbyl palmitate, inhibition of hyaluronic acid synthesis using the FDA‐approved prescription drug 4‐methylumbelliferone (4‐MU) or genetic deletion of hyaluronan synthase genes has been shown to improve glucose homoeostasis and is already being explored for the treatment of obesity and diabetes.^74^ This is particularly salient given the mounting evidence that metabolic dysfunction is an important component of substance use disorders.^75^ However, additional research is needed to clarify the role of hyaluronic acid biology in CUD and to evaluate the potential of pharmacological agents for repurposing as CUD treatments. This could include in vivo investigation of these compounds in suitable animal models of addiction‐associated behaviours, as well as characterising additional evidence of dysregulated hyaluronic acid biology in CUD using resources such as post‐mortem brain. Moreover, follow‐up studies should also be conducted to validate the potential risk genes prioritised in this study including functional investigations.

This study has several limitations. Firstly, we used European‐only summary statistics, and research has shown that predominantly European‐ancestry studies do not translate well to other ancestries and may give an incomplete picture of the genetic underpinnings of CUD. Second, the GWAS was performed using multiple cohorts from the PGC substance use disorders working group, as well as the iPSYCH and deCODE cohorts. Each cohort used different diagnostic criteria, with iPSYCH CUD cases defined using ICD10 codes (F12.1‐12.2) and deCODE cases used DSM‐IIIR, DSM‐IV and DSM‐V criteria for case phenotyping. The cohorts from the PGC substance use disorders working group also had limited homogeneity, with controls for some studies defined as people who simply did not meet criteria for cannabis abuse or dependence, and co‐morbid diagnoses retained in case samples. Therefore, it is crucial that future GWAS of CUD focus predominantly on homogeneity in sample populations.

Finally, while common variant associations with CUD only explain a small amount of phenotypic variance, the effect of risk alleles on molecular traits, like gene expression, are arguably large enough to warrant therapeutic intervention, particularly as these genetics informed targets tend to have a high level of statistical confidence.^76^, ^77^ Larger sample size panels of expression studies to estimate QTLs and GReX, as well as more diverse neurological tissues and cell‐type data, will further increase discovery power in these approaches in future studies.

## AUTHOR CONTRIBUTIONS

Laura A. Greco, William R. Reay and Murray J. Cairns designed the study. Laura A. Greco performed the primary analyses. William R. Reay performed the finemapping and pheWAS. Christopher V. Dayas and Murray J. Cairns supervised the project. Laura A. Greco drafted the manuscript with input from William R. Reay. All authors contributed to the interpretation of the results and the final manuscript.

## CONFLICT OF INTEREST STATEMENT

M.J.C. and W.R.R. are directors at PolygenRx Pty Ltd. The remaining authors declare no competing financial interests.

## Supporting information


**Table S1:** Gene based multivariate association individual cannabis use disorder GWAS.
**Table S2:** Phenome‐wide association study (PheWAS) of the *PDE4B* variant with the highest posterior proability in the 95% credible set – IEUGWASdb.
**Table S3:** Phenome‐wide association study (PheWAS) of the *PDE4B* variant with the highest posterior proability in the 95% credible set ‐ FinnGen release 7.
**Table S4:** TWAS results for cannabis use disorder.
**Table S5:** PWAS results for cannabis use disorder.
**Table S6:** LD‐Score Regression of CUD and 50 biochemical traits.
**Table S7:** Latent Causal Variable (LCV) modelling of CUD and LDSR significant traits.

## Data Availability

The authors confirm that the data supporting the findings of this study are publicly available and accessible online and can be found within the article and its supplementary material. The CUD summary statistics can be found here: 10.6084/m9.figshare.14842692.
